# Changes in brain functional connectivity and muscle strength independent of elbow flexor atrophy following upper limb immobilization in young females

**DOI:** 10.1113/EP091782

**Published:** 2024-06-27

**Authors:** Freddie Seo, Julien Clouette, Yijia Huang, Alexandra Potvin‐Desrochers, Henri Lajeunesse, Frédérike Parent‐L'Ecuyer, Claire Traversa, Caroline Paquette, Tyler A. Churchward‐Venne

**Affiliations:** ^1^ Department of Kinesiology and Physical Education McGill University Montreal QC Canada; ^2^ Centre for Interdisciplinary Research in Rehabilitation of Greater Montreal Montreal QC Canada; ^3^ Division of Geriatric Medicine McGill University Montreal QC Canada; ^4^ Research Institute of the McGill University Health Centre Montreal QC Canada

**Keywords:** brain plasticity, functional magnetic resonance imaging, muscle disuse

## Abstract

Muscle disuse induces a decline in muscle strength that exceeds the rate and magnitude of muscle atrophy, suggesting that factors beyond the muscle contribute to strength loss. The purpose of this study was to characterize changes in the brain and neuromuscular system in addition to muscle size following upper limb immobilization in young females. Using a within‐participant, unilateral design, 12 females (age: 20.6 ± 2.1 years) underwent 14 days of upper arm immobilization using an elbow brace and sling. Bilateral measures of muscle strength (isometric and isokinetic dynamometry), muscle size (magnetic resonance imaging), voluntary muscle activation capacity, corticospinal excitability, cortical thickness and resting‐state functional connectivity were collected before and after immobilization. Immobilization induced a significant decline in isometric elbow flexion (−21.3 ± 19.2%, interaction: *P *= 0.0440) and extension (−19.9 ± 15.7%, interaction: *P* = 0.0317) strength in the immobilized arm only. There was no significant effect of immobilization on elbow flexor cross‐sectional area (CSA) (−1.2 ± 2.4%, interaction: *P *= 0.466), whereas elbow extensor CSA decreased (−2.9 ± 2.9%, interaction: *P* = 0.0177) in the immobilized arm. Immobilization did not differentially alter voluntary activation capacity, corticospinal excitability, or cortical thickness (*P *> 0.05); however, there were significant changes in the functional connectivity of brain regions related to movement planning and error detection (*P *< 0.05). This study reveals that elbow flexor strength loss can occur in the absence of significant elbow flexor muscle atrophy, and that the brain represents a site of functional adaptation in response to upper limb immobilization in young females.

## Abstract (highlights)

- **What is the central question of this study?**
  To what extent does 14 days of upper limb immobilization affect muscle strength, muscle size and neuromuscular function in young females?
- **What is the main finding and its importance?**
  Fourteen days of upper limb immobilization induces a significant decline in muscle strength as well as changes in resting state functional connectivity between brain regions implicated in motor learning and feedback, with little effect on muscle size, corticospinal excitability and voluntary muscle activation capacity. These findings suggest that the nervous system may play a role in the initial decline in muscle function with upper arm immobilization in young females.

## 1 INTRODUCTION

Obligatory periods of skeletal muscle disuse (e.g., due to limb immobilization or bed rest) are known to result in significant strength loss and muscle atrophy (i.e., the loss of muscle mass). Research has largely demonstrated that the rate and magnitude of disuse‐induced strength loss exceeds that of muscle atrophy, suggesting that changes in muscle function cannot be explained exclusively by changes in muscle size (Campbell et al., 2019). Given that the brain is ultimately responsible for the onset of voluntary skeletal muscle contraction, the central nervous system may represent a relevant site of interest for understanding the disproportionate loss of muscle strength compared to size observed in response to disuse. It is well accepted that neural adaptations precede observable muscle hypertrophy during the first several weeks of resistance exercise training in untrained individuals (Pearcey et al., 2021). As such, it is possible that the central nervous system is the first site of adaptation during muscle disuse. In fact, prior studies have revealed that limb immobilization can elicit significant changes in corticospinal excitability within as little as 3 h (Karita et al., 2017), and with longer periods of immobilization, changes in brain structure and activity (Burianova et al., 2016; Garbarini et al., 2019; Langer et al., 2012).

While muscle disuse leads to a decline in both muscle size and strength, a recent systematic review by Campbell and colleagues quantifying changes in muscle size, strength and neuromuscular function in response to limb immobilization in humans reported no significant relationship between the rate of change in muscle size and muscle strength in response to either upper or lower limb immobilization (Campbell et al., 2019). On the other hand, in the upper limb specifically, there was a significant positive relationship between the change in strength and the change in voluntary activation (VA) capacity of the corresponding muscles (Campbell et al., 2019); but studies are limited. Unlike many of the muscles in the lower limb, upper limb muscles exhibit a greater density of corticospinal projections and are not responsible for weight bearing (Brouwer & Ashby, 1990). It is therefore possible that muscle atrophy plays a less significant role in disuse‐related strength loss in the upper compared to the lower limb. In support of this notion, the disuse‐induced decline in relative muscle strength has been reported to be similar between the upper and lower limbs (1.3%/day), while the decline in muscle size in the upper limbs (0.2%/day) is half that of the lower limbs (0.4%/day) (Campbell et al., 2019). However, upper limb immobilization studies to date have lacked the use of criterion measures, namely, isokinetic dynamometry and either magnetic resonance imaging (MRI) or computed tomography for muscle strength and size assessment, respectively (Campbell et al., 2019). To our knowledge, no study to date has employed current standards of measurement for the assessment of both muscle size and strength in response to upper limb immobilization.

There is some evidence that females may be more susceptible to the deleterious effects of muscle disuse on muscle function than males (Deschenes et al., 2012). Females have been demonstrated to lose more relative muscle strength in response to lower limb immobilization compared to males despite no differences in relative muscle atrophy (Deschenes et al., 2009, 2012; Yasuda et al., 2005). This observation may be attributed to differences in neuromuscular plasticity, as it was additionally found that females lost greater than four times the amount of surface electromyographical (EMG) activity recorded during maximal voluntary knee extension compared to males following lower limb immobilization (Deschenes et al., 2009). In contrast, a study exploring sex differences in response to upper limb suspension revealed a similar decline in relative strength between males and females (Miles et al., 2005). However, only males experienced a significant reduction in elbow flexor muscle volume, suggesting that mechanisms apart from muscle atrophy were responsible for strength loss in females (Miles et al., 2005). Despite this, females currently represent only ∼24% of the participants included among limb immobilization studies measuring changes in both muscle size and strength (Campbell et al., 2019). As such, the effect of upper limb immobilization on skeletal muscle size and strength in females is less known.

The purpose of this study was to evaluate changes in muscle strength, muscle size, VA, corticospinal excitability, cortical thickness and resting‐state functional connectivity (rs‐FC) in response to 14 days of upper limb immobilization in young females using current standards of measurement. We hypothesized that immobilization would result in a significant decrease in muscle strength, muscle size and VA after 14 days exclusively in the immobilized limb. We additionally hypothesized that immobilization would induce an early transient decrease in corticospinal excitability in the immobilized arm at 24 h post‐immobilization. A hypothesis‐driven analysis of rs‐FC outcomes was developed and conducted by Clouette et al. (2024). It was hypothesized that sensorimotor regions corresponding to the immobilized limb would segregate from the remainder of the sensorimotor network, and that the primary motor cortex (M1) would undergo significant changes in connectivity. Cortical thickness data were obtained for exploratory purposes.

## 2 METHODS

### 2.1 Ethics approval

This study was approved (approval no. A01‐M01‐21A) by the Faculty of Medicine and Health Sciences Institutional Review Board at McGill University and was carried out in accordance with the *Declaration of Helsinki* of 1975 as revised in October 2013. All participants were informed by a study investigator regarding the purpose of the study, the procedures involved, and the possible risks associated with participation in the study before providing informed written consent. The study was registered at: https://clinicaltrials.gov/study/NCT05115643.

### 2.2 Participants and sample size determination

Twelve right‐hand dominant females (age: 20.6 ± 2.1 years; height: 164 ± 6.3 cm; weight: 58.4 ± 3.2 kg; body mass index: 21.5 ± 3.2 kg/m^2^; means ± SD) were included in the present study. All participants reported having a regular menstrual cycle. Three of the 12 participants (25%) reported engagement in deliberate exercise training or sports (mean ± SD (*n* = 12): 1.0 ± 2.0 h/week). None of the participants engaged in structured resistance exercise training ≥3 months prior to commencing the study. Participants were excluded from the study if they met any of the following criteria: use of tobacco, pregnancy, history of brain trauma, neurological disease, movement disorder, mental illness, peripheral nerve damage, use of certain medications or supplements known to affect protein metabolism (e.g., corticosteroids, non‐steroidal anti‐inflammatory drugs, prescription strength acne medications, creatine, fish oil), or contraindications to MRI or transcranial magnetic stimulation (TMS). Accounting for the unilateral within‐participant study design, in which the non‐immobilized limb is used as an internal co‐temporal control, it was determined using G*Power software (version 3.1.9.7) that 12 volunteers would be sufficient to detect a medium effect size (Cohen's *f* = 0.25) with an α of 0.05 and a power level of at least 0.9 for isometric muscle strength and muscle size.

### 2.3 Overview of the experimental design

The study followed a within‐participant, unilateral design comparing the immobilized versus non‐immobilized arm of each participant. Upon providing informed consent, participants provided baseline measures indicative of their general health status and underwent a familiarization session with the isokinetic dynamometer used for strength testing (Biodex 4 ProTM, Biodex Medical Instruments, Shirley, NY, USA). This included a basic medical questionnaire, as well as measurement of anthropometrics (height and weight), body composition (by dual‐energy X‐ray absorptiometry; GE Healthcare, Madison, WI, USA), and resting heart rate and blood pressure (Omron Healthcare Co., Ltd. 10 series, Model BP786CANN, Kyoto, Japan). Familiarization with the Biodex involved performing one contraction of each strength parameter measured on each arm. During the experimental period, each participant's left arm was immobilized for 14 consecutive days. Outcome measures were assessed pre‐ and post‐immobilization in the following order: bilateral elbow flexor and extensor muscle size; anatomical and resting‐state functional MRI (rs‐fMRI) scans of the brain; serum hormone concentrations (17β‐oestradiol and progesterone); bilateral corticospinal excitability of the biceps brachii; bilateral VA of the biceps brachii; and bilateral elbow extensor and flexor muscle strength. To evaluate the short‐term effects of the intervention on the nervous system, corticospinal excitability of the biceps brachii was additionally measured at 24 h following upper limb immobilization. In consideration of potential confounding by changes in dietary protein intake, participants recorded their food intake using standardized forms during the first 2 days before, and last 2 days of the immobilization period. Dietary intake data were analysed using commercially available software (Food Processor version v.11.7; ESHA Research, Salem, OR, USA). Participants were asked not to engage in any form of moderate to vigorous physical activity from the 48‐h period prior to the pre‐immobilization testing visit until the end of the study. They were additionally asked not to consume any alcohol throughout the immobilization period, and not to consume any caffeine 24 h prior to all study visits. All participants received $300 CAD following the post‐immobilization evaluation visit as compensation for any expenses incurred as a result of participating in the research.

### 2.4 Intervention

#### 2.4.1 Upper arm immobilization

**FIGURE 1:**
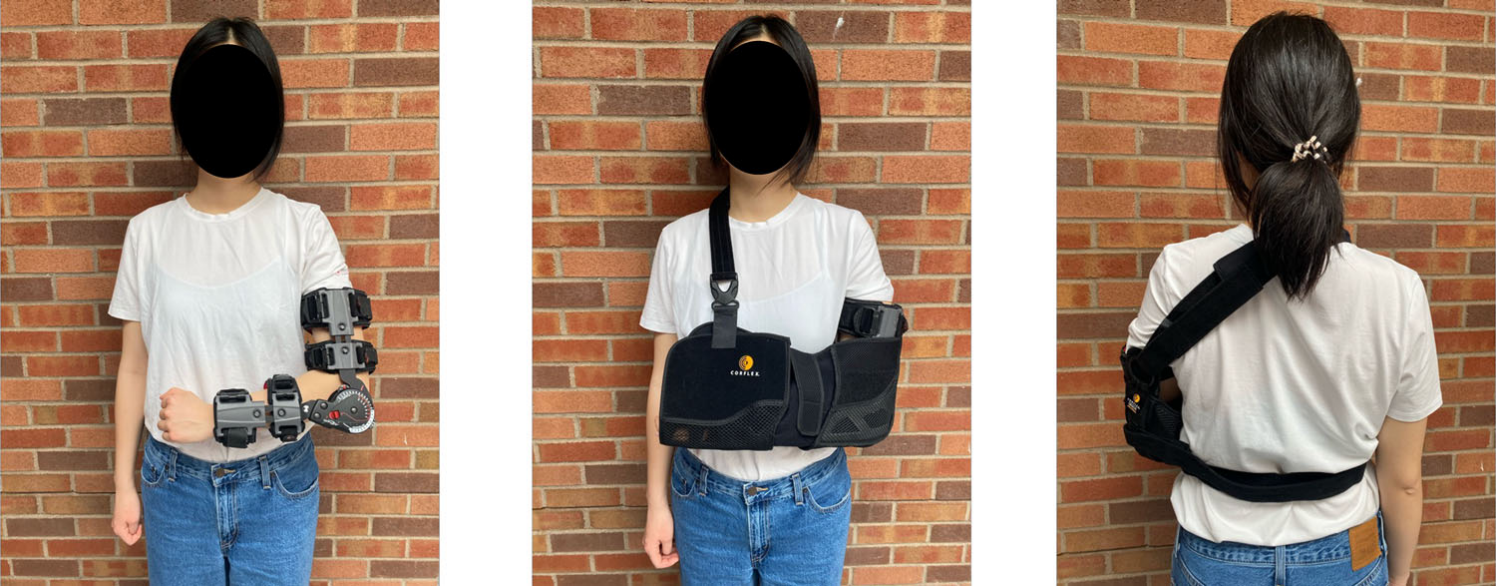
Representative image depicting the unilateral upper limb immobilization model. A telescoping arm brace was applied to the non‐dominant (left) arm fixed at 90° elbow flexion. A sling was applied over the telescoping arm brace to prevent shoulder flexion.

Following pre‐immobilization testing, participants underwent immobilization of their left arm using a telescoping arm brace (Donjoy, Lewisville, TX, USA) fixed at 90° elbow flexion and a sling (to prevent shoulder flexion). To ensure participant compliance to the immobilization, zip ties were placed around the telescoping arm brace to prevent its removal. Unique code words were written in ink by a study investigator on each zip tie so that any zip ties removed could not be replaced without the investigator's knowledge. The telescoping arm brace was worn at all times, including during sleep and bathing. Participants were allowed to remove the sling momentarily when bathing, changing clothes and sleeping. Reusable waterproof brace covers were provided to participants to prevent the brace from getting wet during bathing. An image depicting the immobilization model is shown in Figure 1.

### 2.5 Outcome measures

#### 2.5.1 Muscle size

All MRI scans were performed using a Siemens 3T Trio Scanner (Siemens, Knoxville, TN, USA) at the McConnell Brain Imaging Centre (Montreal Neurological Institute, Montreal, Canada). Cross‐sectional images of the upper arm muscles were obtained using a T2‐weighted imaging protocol (acquisition time = 3:36 min each arm; echo time = 9.6 ms; repetition time = 3300 ms; matrix 256 × 256 × 44 slices; 0.8 × 0.8 × 5 mm). Two scans were performed on each arm, divided spatially using a radiological marker placed at the midpoint between the acromion process and the olecranon. Size of the right and left elbow flexor and extensor muscle groups were defined by their cross‐sectional area (CSA) and volume using the open‐source computer software MRtrix3 (https://www.mrtrix.org/). Beginning at the proximal edge of the humerus, the borders of the muscle were manually traced by a sole blinded investigator (Y.H.) to obtain measures of cross‐sectional area (CSA) within individual slices until the elbow joint was visible. As there was no gap in between slices, volume was calculated by summating the individual CSAs and multiplying the result by the slice thickness. To assess the degree of intra‐rater measurement error, muscle size measurements were repeated for three participants, with a minimum of 4 days between measurements. The coefficient of variation (CV) was calculated by dividing the standard deviation of the two measurements by the mean.

#### 2.5.2 Cortical thickness

Structural images were obtained via T1‐weighted anatomical MRI (acquisition time = 6:44 min; echo time = 7.7 ms; inversion time = 900; repetition time = 2300 ms; 0.8 mm^3^ isotropic; flip angle = 9°). MRI data were processed through the automated image‐processing pipeline CIVET (version 2.1.1, Montreal Neurological Institute) (Lepage et al., 2017). The CIVET pipeline registers the T1 anatomical image to the icbm152nl using a nine‐parameter linear registration (0.5 mm voxels). Tissue is then classified into white matter (WM), grey matter (GM) and cerebral spinal fluid. The WM and GM boundaries are extracted and fitted with 81,920 polygons. The difference between WM and GM surfaces is calculated as cortical thickness. The following parameters were additionally used to process the data: N3 distance of 125 mm (to correct nonuniformity artefacts), Tlink as the measure of cortical thickness, and 30 mm kernel size (for smoothing). Quality control of the CIVET output was done manually for each participant through visual examination of the display borders of cortical images.

#### 2.5.3 Functional connectivity

Functional connectivity (FC) was measured using blood oxygen level‐dependent (BOLD) MOSAIC rs‐fMRI (acquisition time = 7:07, 700 volumes, voxel size = 3 mm^3^ isotropic, echo spacing = 0.54 ms, 48 slices, flip angle = 50°). During the rs‐fMRI, participants were asked to lie still with their eyes closed, remain awake and not to think about anything in particular. Preprocessing, analysis, and statistical computations were performed using a resting‐state pipeline developed by the Center for Research on Brain, Language and Music (www.crblm.ca), which integrated the use of FSL 5.0.8 (FMRIB Software Library, Oxford, UK) and MATLAB 2018b (MathWorks, Natick, MA, USA) software packages as employed in previous studies from our group (Potvin‐Desrochers et al., 2023, 2019). A seed‐to‐voxel analysis was used to measure FC. Seeds were selected a priori and were created using masks in the MNI space. Seed selection was based on pre‐existing knowledge of brain regions involved in motor control as well as evidence on the effect of limb immobilization on brain structure and function. Seeds included in the fMRI analysis were: M1 and S1 representations of the upper arm (Newbold et al., 2020); supplementary motor area (SMA) (Garbarini et al., 2019); cerebellar lobules implicated in upper arm control (lobules VI, VIIb, VIIIa, VIIIb, IX) (Mottolese et al., 2013); globus pallidus (including the interna and externa nuclei) and putamen; thalamus (including the following nuclear divisions of the motor thalamus: ventral lateral, ventral anterior, ventral posterolateral, ventral posteromedial) (Maldjian et al., 2003); and ventral (vPMC) and dorsal (dPMC) premotor cortex (Garbarini et al., 2019). Further details regarding the fMRI procedures and analysis can be found in Clouette et al. (2024).

#### 2.5.4 Blood collection and ovarian hormone analysis

Blood samples were analysed for serum concentrations of progesterone (nmol/L) and 17β‐oestradiol (pmol/L) to confirm whether their levels fluctuated significantly between pre‐ and post‐immobilization measurement points (Clinical Biochemistry Laboratory of the McGill University Health Centre). This is due to the possibility that normal fluctuations in ovarian hormones throughout the female menstrual cycle alter day‐to‐day corticospinal excitability (Smith et al., 2002). Eight millilitres of blood was collected in a serum vacutainer (BD Vacutainer SST Blood Collection Tubes; Becton Dickinson, Mississauga, ON, Canada), centrifuged at 1000 *g* after 30 min, and stored at −80°C in 1.5 mL Eppendorf tubes until analysis.

#### 2.5.5 Transcranial magnetic stimulation

TMS was administered using a Super Rapid^2^ TMS system (Magstim Company, Whitland, UK) connected to a dome‐shaped coil (Jaltron LLC, Lexington, MA, USA). With the participant seated comfortably, the TMS coil was positioned tangentially to the participant's right or left M1 at a 45° angle from the mid‐sagittal line to stimulate the motor region controlling the upper arm muscles. EMG activity of the biceps brachii was recorded using disposable surface electrodes (Biopac Systems, Inc., Goleta, CA, USA) positioned in a belly‐tendon montage. EMG data were processed by a Biopac MP150 acquisition system (Biopac Systems, Inc.), sampled at 10 kHz on a 16‐bit analog‐to‐digital board, and amplified and bandpass filtered at 10–5000 Hz. The ‘hotspot’, defined as the stimulation site yielding the greatest EMG response in the biceps brachii, was located using a frameless stereotaxic neuronavigational software (Brainsight, Rogue Research Inc., Montreal, Canada) outfitted with a three‐dimensional reconstruction of the participant's brain obtained from their T1‐weighted anatomical MRI scan.

Corticospinal excitability of the biceps brachii was inferred using both resting motor threshold (RMT) and stimulus–response (SR) curves. RMT of the biceps brachii was defined as the minimum TMS intensity required to elicit a motor evoked potential (MEP) of 0.05 mV in ≥10 out of 20 trials (Rossini et al., 2015). Data for SR curves was acquired through the administration of 10 pulses at various intensities separated by increments of 10% of maximum stimulator output (%MSO, range = 30–90), in a randomized order. When necessary, additional TMS stimuli were administered to replace trials in which muscle activity was evident immediately before or during a MEP. Individual MEPs were excluded during analysis if they were classified as an outlier using the Tukey method among the 10 pulses administered at a given %MSO (Kannan et al., 2015). A total of 58 of 5280 (1.1%) outliers were excluded across the analysis of all TMS outcomes. Individual SR curves were constructed by fitting mean peak‐to‐peak MEP amplitudes at each TMS intensity against a sigmoidal curve using the Solver function of Microsoft Excel with the following equation:

$$\mathrm{MEP}\;\mathrm{amplitude}\;\left(\mathrm{mv}\right)\;=\frac{\mathrm{ME}P_{\max}\;}{1+\;e^{\left(\frac{S_{50}\;-S}{k}\right)}}$$

where *S* is the %MSO, *S*_50_ is the %MSO at which 50% of the maximum MEP amplitude is observed (i.e., the inflection point of the curve), and *k* is the slope of the tangent passing through the inflection point. A coefficient of determination (*R* ^2^) > 0.7 was considered an acceptable fit (Potter‐Baker et al., 2016). Excitability was quantified as the area under the curve (AUC), which was calculated using trapezoidal integration.

Two of the 12 participants were not able to tolerate some of the higher TMS intensities (70%–90%MSO). Appropriate measures, such as replacing these intensities with stimuli at lower %MSOs were undertaken so that a relationship between stimulus and response could still be evaluated. Certain individuals were also found to have low RMTs (<30%MSO). For these participants, an additional 10 stimuli at 20%MSO were administered for both arms to extract the portion of the SR curve at which MEP amplitudes of zero are observed. The array of intensities was kept consistent between measurement sessions within participants in which the TMS procedure had to be adapted.

#### 2.5.6 Voluntary activation capacity

VA of the biceps brachii was measured in both arms using the twitch interpolation technique via peripheral muscle stimulation (Digitimer, Welwyn Garden City, UK) (Todd et al., 2004). Torque data were obtained using the same isokinetic dynamometer used to measure muscle strength, which was processed and recorded using an analog‐to‐digital converter (Micro 1401, CED, Cambridge, UK) connected to a laptop running Spike2 software (version 10, CED). Two, 5 × 10 cm oval neurostimulation electrodes (Axelgaard Manufacturing Co., Lystrup, Denmark) were used to administer electrically evoked doublets to the biceps brachii muscle (100 μs pulse width; 10 ms interstimulus interval). The anode was placed on the distal tendon of the biceps, over the antecubital fossa, whereas the cathode was moved around the muscle belly of the biceps until the site of stimulation yielding the highest evoked elbow flexion torque was located. Stimuli were administered during maximal isometric elbow flexion contractions at 120% of the intensity yielding the highest possible elbow flexion torque at rest. The superimposed doublet was triggered manually once the participant reached a plateau in torque; the resting doublet was triggered once torque returned to baseline level, approximately 3–5 s later. Trials in which the participant and/or the torque data indicated that the electrical stimulus was not administered near the participant's maximal achievable torque were discarded. A minimum of three trials were performed on each arm, and the highest value was recorded for analysis. VA was calculated as a percentage within Spike2 by comparing the additional elbow flexion torque produced by suprathreshold stimulation of the biceps brachii during maximal contraction, to the torque evoked at rest using the following equation:

$$\mathrm{Voluntary}\;\mathrm{activation}\;\;\left(\%\right)=\left(1-\;\frac{\mathrm{superimposed}\;\mathrm{twitch}}{\mathrm{resting}\;\mathrm{twitch}}\right)\;\times \;100$$

VA was determined to be 100% for trials in which the stimulus was administered at peak torque but failed to produce a noticeable superimposed twitch.

#### 2.5.7 Muscle strength

Muscle strength was measured as maximal voluntary isometric and isokinetic (maximum velocity of 120°/s) contraction in both arms. Participants performed three 5‐s maximal isometric contractions to measure the strength of the elbow extensor, then flexor muscles. The elbow joint was fixed at 90° and aligned with the machine's axis of rotation. For isokinetic measurements, participants were instructed to contract as hard and as fast as possible through their full range of motion at the elbow joint. Verbal encouragement was provided during all strength tests. All contractions were separated by 90 s of rest. The highest peak torque achieved among the three trials for each contraction type was recorded as muscle strength.

### 2.6 Statistical analysis

Bilateral elbow flexor and extensor muscle size (CSA and volume), bilateral elbow flexor and extensor muscle strength (isometric and isokinetic), and RMT were analysed with a two‐way repeated measures ANOVA, with time (pre‐ vs. post‐immobilization) and arm (immobilized vs. non‐immobilized) as within‐participant factors. For RMT, an additional level was added to time as a within‐participant factor to include measurements at 24 h post‐immobilization. If a significant time × arm interaction was found, Bonferroni‐adjusted pairwise comparisons were performed. Serum ovarian hormone concentrations pre‐ and post‐immobilization and dietary protein intake (absolute (g) and relative (g/kg body weight/d)) during the first 2 days before, and last 2 days of the immobilization period, were compared using Student's paired‐sample *t* test. For TMS SR curve analysis, AUC was expressed as percentage change from baseline and analysed using a paired‐sample *t*‐test due to high variability in baseline excitability within our sample. As many individuals began with VA levels near or at 100%, VA was also expressed as a percentage change from baseline and analysed using a paired‐sample *t*‐test. Assumptions of the ANOVA models were assessed using Mauchley's test and visual inspection of Q‐Q plots of the residuals. If a significant Mauchley's test was determined, the Greenhouse–Geisser correction factor was used to adjust the degrees of freedom accordingly. Outliers in the data were identified using the Tukey method. For the above analyses, statistical calculations were performed using the SPSS Statistics, Version 26 (IBM Corp., Armonk, NY, USA). A *P*‐value <0.05 was considered statistically significant. All data are expressed as means ± standard deviation (SD), along with individual participant data where feasible.

For the analysis of brain cortical thickness, the SurfStat toolbox (http://www.math.mcgill.ca/keith/surfstat/) operating in MATLAB version 9.13.0.2105380 (R2022b) was used (Worsley et al., 1996). A mixed effects linear model was fitted to vertex‐wise data with variables of interest as time point (Pre–Post, fixed effects) and subject (random effects). *t*‐Value maps were computed in SurfStat for the Pre–Post contrast and corrected *P*‐values for the model were obtained using the random field theory (RFT) controlling for the probability of reporting false positives (Worsley et al., 1999). A region of interest (ROI) analysis was conducted using the combination of three clusters of the left hemisphere identified by Langer et al. (2012) centred at MNI coordinates (−35, −21, 53), (−58, −18, 46), and (−50, −21, 57) with 10 mm radius each; the same coordinates were flipped to analyse the right hemisphere ROI.

For the rs‐FC statistical analysis, individual FC maps were obtained using a rs‐FC regression analysis in native space which included nuisance variables (cerebrospinal fluid, white matter (WM), global signal, motion outlier volume masks and motion parameters). A mixed‐effect mode using Bayesian modelling scheme in FLAME, FSL was used to determine within‐subject pre‐ to post‐immobilization differences (Woolrich et al., 2004). Correction for multiple comparisons was performed using a cluster threshold of *Z* > 3.1, and a cluster significance of *P *< 0.05 (Worsley, 2001).

## 3 RESULTS

### 3.1 Participant dietary protein intake

There was no significant change in daily absolute (66 ± 18 to 57 ± 27 g; *P* = 0.174) and relative (1.2 ± 0.3 to 1.0 ± 0.5 g/kg body weight/day; *P* = 0.201) protein intake between the period before and during immobilization.

### 3.2 Ovarian hormones

Serum 17β‐oestradiol (218.8 ± 239.9 to 346.8 ± 406.3 pmol/L; *P* = 0.214) and progesterone (5.0 ± 3.9 to 5.9 ± 5.0 nmol/L; *P* = 0.452) concentration was not significantly different between pre‐ and post‐immobilization testing visits.

### 3.3 Muscle strength

**FIGURE 2:**
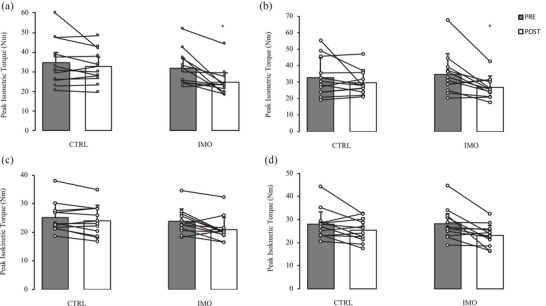
Peak torque during isometric elbow flexion (a), isometric elbow extension (b), isokinetic elbow flexion (c), and isokinetic elbow extension (d) before (PRE) and after 14 days (POST) of non‐dominant upper limb immobilization (sample sizes for a–d: 12 females). Values are means ± SD. *Significantly different from baseline within respective arm (a: interaction *P* = 0.0440; b: interaction *P *= 0.0317). CTRL, non‐immobilized arm; IMO, immobilized arm.

Changes in all strength variables in response to the immobilization are displayed in Figure 2. Certain strength parameters failed to meet the assumption of normality required for ANOVA. An individual with high baseline strength compared to the rest of the sample was identified as an outlier using the Tukey method. Upon removal of this outlier, all strength variables satisfied the conditions of normality. Statistical tests were performed both with and without the outlier to confirm whether the interpretation of the results remained consistent. The results reported below and in Figure 2 are with the outlier included in the analysis.

There was a significant time × arm interaction effect for both isometric elbow flexion (interaction: *P* = 0.0440 and extension (interaction: *P *= 0.0317) torque. Bonferroni‐adjusted pairwise comparisons indicated that strength decreased significantly following the intervention in the immobilized arm (flexion: 32.0 ± 8.7 to 24.7 ± 7.0 N m; *P* = 0.00418; extension: 34.7 ± 12.5 to 26.7 ± 6.9 N m; *P* = 0.00471) but did not change in the non‐immobilized arm (flexion: 34.9 ± 11.7 to 32.8 ± 8.5 N m; *P* = 0.214; extension: 32.7 ± 12.0 to 29.6 ± 7.6 N m, *P* = 0.254). The mean relative change in isometric strength in the immobilized arm was −21.3 ± 19.2% and −19.9 ± 15.7% for elbow flexion and extension, respectively. There was no significant interaction effect for isokinetic elbow flexion (interaction: *P* = 0.151) or extension torque (interaction: *P* = 0.114). Statistical interpretations of the strength results remained consistent when the outlier was excluded from the analysis.

### 3.4 Muscle size

**FIGURE 3:**
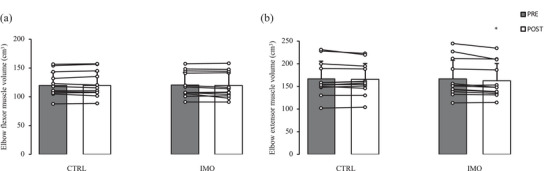
Elbow flexor (a) and extensor (b) muscle volume before (PRE) and after 14 days (POST) of non‐dominant upper limb immobilization (sample sizes for a, b: 12 females). Values are means ± SD. *Significantly different from baseline within respective arm (b: interaction *P *= 0.0429). CTRL, non‐immobilized arm; IMO, immobilized arm.

Changes in elbow flexor and extensor muscle volume are displayed in Figure 3. Mean CVs for repeated muscle size assessments were 0.70 (flexor volume), 0.47 (extensor volume), 0.76 (flexor CSA), and 0.67 (extensor CSA). There were no baseline differences between arms for all measures of muscle size. There were no significant effects of the immobilization on elbow flexor CSA (immobilized: 10.9 ± 2.3 to 10.9 ± 2.3 cm^2^; non‐immobilized: 10.6 ± 2.1 to 10.6 ± 2.2 cm^2^; interaction: *P* = 0.466). There was a significant time × arm interaction effect for elbow flexor volume (interaction: *P* = 0.0345); however, Bonferroni‐adjusted pairwise comparisons did not reveal any significant differences between means regardless of whether the data were stratified by arm or time point of measurement (immobilized: 120.5 ± 21.8 to 119.2 ± 22.5 cm^3^; *P* = 0.114; non‐immobilized: 119.4 ± 23.1 to 119.5 ± 24.1 cm^3^, *P* = 0.917). There were significant interaction effects for both elbow extensor CSA (interaction: *P* = 0.0177) and volume (interaction: *P* = 0.0429), such that they decreased significantly in the immobilized arm (CSA: 15.3 ± 3.8 to 14.8 ± 3.5 cm^2^; *P* = 0.0111; volume: 166.8 ± 41.2 to 162.1 ± 38.3 cm^3^; *P* = 0.0111) but did not change in the non‐immobilized arm (CSA: 15.3 ± 3.5 to 15.2 ± 3.3 cm^2^; *P* = 0.768; volume: 167.0 ± 38.3 to 166.0 ± 34.9 cm^3^; *P* = 0.467). The mean relative change in elbow extensor CSA and volume in the immobilized arm from pre‐ to post‐immobilization was −2.9 ± 2.9% and −2.5 ± 2.5%, respectively.

### 3.5 Voluntary activation capacity

**FIGURE 4:**
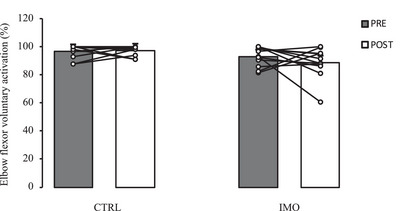
Voluntary activation capacity (VA) of the biceps brachii before (PRE) and after 14 days (POST) of non‐dominant upper limb immobilization (sample size: 11 females). Values are means ± SD. CTRL, non‐immobilized arm; IMO, immobilized arm.

VA data were available in 11 of the 12 participants. There was no significant difference in the relative change in VA between arms following the intervention (Figure 4; −4.3 ± 14.1% in immobilized vs. 0.4 ± 5.9% in non‐immobilized arm, *P* = 0.354).

### 3.6 Corticospinal excitability

**FIGURE 5:**
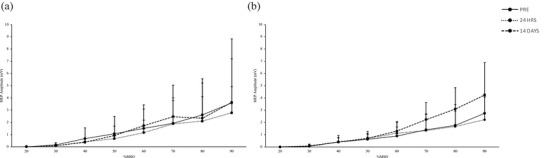
Stimulus–response curve as represented by peak‐to‐peak motor evoked potential (MEP) amplitudes from 20% to 90% of maximum stimulator output (%MSO) using transcranial magnetic stimulation before (PRE), 24 h after (24 HRS), and 14 days after (14 DAYS) non‐dominant upper limb immobilization. (a) Non‐immobilized arm; (b) immobilized arm. Values are means + SD. Sample sizes for a, b: 12 females, within‐subject design.

A visual representation of the SR curves at pre‐, 24 h post‐, and 14 days post‐immobilization is displayed in Figure 5. Mean baseline RMT was approximately 31 ± 7% and 32 ± 7%MSO in the non‐immobilized and immobilized arms, respectively, with no differences between arms (arm: *P* = 0.275). There were no significant effects detected for RMT throughout the immobilization period (time: *P* = 0.463; interaction: *P* = 0.544). The mean relative change in AUC from baseline at 24 h post‐immobilization was −3.2 ± 42.7% and 30.4 ± 70.7% in the non‐immobilized and immobilized arm, respectively, with no significant differences between arms (*P* = 0.173). Two outliers that skewed the data away from a normal distribution were identified at the 14 days post‐immobilization time point, one showing a 242.8% increase in AUC in the non‐immobilized arm, and another with a 645.3% increase in the immobilized arm. Upon removal of these two outliers, the data met the conditions of normality. The adjusted mean relative change in AUC from baseline at 14 days post‐immobilization was −2.7 ± 54.5% and 58.8 ± 90.6% in the non‐immobilized and immobilized arm, respectively (*n* = 10). A paired‐samples *t*‐test revealed no significant difference in the mean relative change in AUC between the immobilized versus non‐immobilized arm (*P* = 0.0834, *n* = 10). To account for the removal of data points, we also performed the non‐parametric Wilcoxon signed‐rank test on the full sample; using this test, there was no significant difference between the mean relative change in AUC between arms (*P* = 0.0994, *n* = 12). The mean relative change from baseline in AUC at 14 days post‐immobilization was 21.9 ± 86.5% and 119.4 ± 189.1% in the non‐immobilized and immobilized arm, respectively (*n* = 12).

### 3.7 Resting‐state functional connectivity

**FIGURE 6:**
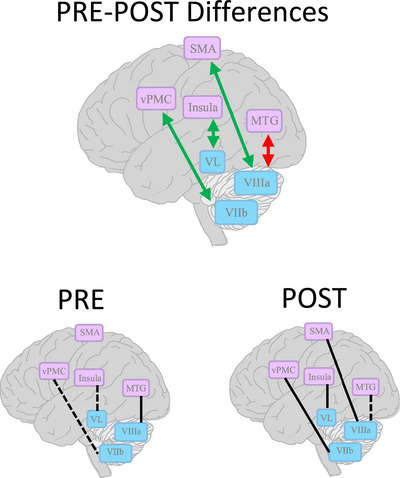
Schematic representation of changes in rs‐FC (top panel) following 14 days of non‐dominant upper limb immobilization (sample size: 12 females), as detailed in Clouette et al. (2024). Green and red arrows represent an increase and decrease in rs‐FC, respectively, after upper limb immobilization. Mean rs‐FC before and after immobilization is presented in the left and right bottom panels, respectively. Dashed lines represent a negative rs‐FC and continuous lines represent a positive rs‐FC. Blue boxes represent the seeds that yield a significant PRE–POST change in rs‐FC with its cluster (purple boxes). SMA, supplementary motor area; vPMC, ventral premotor cortex; VL, ventrolateral nucleus of the thalamus; MTG, middle temporal gyrus; VIIIa, cerebellar lobule VIIIa; VIIb, cerebellar lobule VIIb.

A detailed report of the fMRI results, including visual and tabular representations of all significant findings, results of a secondary analysis using a less stringent threshold of *Z* > 2.6, and correlations between fMRI and behavioural outcomes can be found in Clouette et al. (2024). In summary, there was a decrease in connectivity between the immobilized (left) cerebellar lobule VIIIa and the left middle temporal gyrus (MTG) from a positive connectivity before immobilization, to no connectivity at 14 days post‐immobilization (*P* (corrected) = 0.002). There was an increase in functional connectivity between the immobilized (left) cerebellar lobule VIIIa and the bilateral SMA such that these regions were not functionally connected pre‐immobilization, but expressed a positive connectivity 14 days post‐immobilization (*P* (corrected) = 0.010). A shift from negative to positive connectivity was observed between (1) the cerebellar region of the non‐immobilized (right) lobule VIIb and the non‐immobilized (left) vPMC (*P* (corrected) = 0.036); as well as (2) the immobilized (right) ventrolateral nucleus of the thalamus (VL) and the right posterior insula (*P* (corrected) = 0.030). Significant changes in functional connectivity from pre‐ to post‐immobilization are summarized in Figure 6.

### 3.8 Cortical thickness

There was no change in global cortical thickness following immobilization (PRE: 3.26 (SD 0.10) mm vs. POST: 3.25 (SD 0.07) mm, no significant cluster *P *= 0.232 RFT corrected). Mean cortical thickness within the left and right ROIs also did not change following immobilization (mean thicknesses within the left hemisphere ROIs were PRE: 2.84 (SD 0.15) mm vs. POST: 2.89 mm (SD 0.12), *P* = 0.127; and for the right hemisphere ROIs PRE: 2.81 (0.14) mm vs. POST: 2.84 (0.14) mm, *P *= 0.058).

## 4 DISCUSSION

This study demonstrated that elbow flexor strength loss in response to 14 days of upper limb immobilization can occur in the absence of significant elbow flexor muscle atrophy in young females, and that the brain may represent a site of functional adaptation in response to muscle disuse as highlighted by changes in rs‐FC. To our knowledge, this is the first study of upper limb immobilization in which current standards of measurement for assessing changes in muscle strength and muscle size were simultaneously employed.

As anticipated, immobilization induced a substantial decline in isometric muscle strength of the elbow flexors (−21.3%) and extensors (−19.9%) within 14 days. This translates to a decline in strength of −1.5%/day (elbow flexors) and −1.4%/day (elbow extensors), respectively, which is marginally higher compared to other studies of elbow immobilization using a brace/cast model (elbow flexion: −0.9 to −1.3%/day; extension: −0.6 to −1.3%/day) (Karolczak et al., 2009; Vaughan, 1989; Yue et al., 1997). This may partially be due to the relatively short duration (14 days) of upper arm immobilization in the present study, as immobilization‐induced adaptations are thought to occur most rapidly during the early (first 7 days) disuse period, after which the rate of change plateaus (Campbell et al., 2019). Another notable finding in the present study was that immobilization did not result in a significant decline in isokinetic muscle strength in the elbow flexors or extensors. This may be due to the velocity of contraction, as not only is it well known that concentric force decreases with increasing contraction velocity, but isokinetic elbow flexion contraction at 120°/s has specifically been demonstrated to achieve a submaximal level of integrated EMG activity, work and power relative to maximum (Barnes, 1980). The present findings also agree with results from a study by MacLennan and colleagues, in which isokinetic knee extension strength decreased at a contraction velocity of 180°/s, but not 360°/s in young females in response to 2 weeks of knee immobilization (MacLennan et al., 2021). It is therefore possible that immobilization has a more profound impact on isometric versus isokinetic strength, thus highlighting that quantifying functional loss in response to muscle disuse may depend on the type of evaluation used.

In contrast to our hypothesis, 14 days of upper limb immobilization induced via an elbow brace and sling did not significantly reduce elbow flexor muscle size. It has previously been reported that elbow flexor muscle volume decreased in response to 4 weeks of elbow cast immobilization by ∼11% (Yue et al., 1997), and so we anticipated elbow flexor atrophy to be at least half this amount. However, findings from the present study are consistent with those of Miles and colleagues, who reported that 3 weeks of upper arm suspension induced a statistically significant decline in elbow flexor volume in untrained males, but not females (mean relative change in females = −1.4%) (Miles et al., 2005). Prior to the present study, changes in muscle size with elbow immobilization had not been evaluated using MRI in a study as short as 14 days, let alone in a sample including only females. It is therefore possible that the upper arm muscles of females are less susceptible to disuse‐induced muscle atrophy; however, further research on sex‐based differences in response to muscle disuse are required to address this.

It is now well established that individual muscles exhibit differing degrees of atrophy in response to disuse (Bass et al., 2021), and that on average the decline in muscle size in the upper limbs is ∼50% less than that of the lower limbs in response to immobilization (0.2%/day vs. 0.4%/day) (Campbell et al., 2019). Although we did not observe significant atrophy in the elbow flexors following immobilization, there was a statistically significant decrease in both the CSA and volume of the elbow extensors. This is a notable finding given that the relative change in muscle volume was approximately two times greater in the elbow extensors compared to the flexors, yet the change in isometric strength between the muscle groups was similar. However, given the relatively small size of the elbow extensor muscle group in the female participants, the mean relative change of −2.5% observed in the present study may not be clinically significant. With a previously determined linear regression equation derived from the relationship between elbow extensor torque and muscle volume, we estimate that the mass lost from the elbow extensor muscle group in the present study would correspond to a ∼0.6 N m reduction in isometric torque (Fukunaga et al., 2001), which falls within the absolute measurement error of the isokinetic dynamometer used in the present study (Brookshaw et al., 2020). Nonetheless, these results further suggest that, in the context of limb immobilization, there is little relationship between strength loss and muscle atrophy (Campbell et al., 2019). Though we observed that the elbow extensors atrophied to a greater extent compared to the flexors, the opposite was found in a previous study by Yue et al. (1997). Due to the lack of studies on disuse atrophy in the upper arm, there is no consensus on how the rate of muscle loss compares between the elbow flexors and extensors with immobilization. Perhaps the time course of immobilization‐induced muscle atrophy differs between the elbow flexors and extensors such that the rate of muscle loss plateaus earlier in the extensors, as Yue et al. observed a relative decline in elbow extensor muscle size similar to that found in our study (−1.7%), despite having an immobilization period that was twice as long as ours. Differences in methodological design may also explain the discrepancies between the two studies. For instance, the majority of the participants in the study by Yue et al. were males. As noted earlier, a study comparing the physiological responses to 3 weeks of upper arm suspension between the sexes found significant elbow flexor atrophy in males, but not females (Miles et al., 2005). Additional studies are required to clearly define the susceptibility of different upper limb muscles to disuse atrophy in both sexes.

In the present study, there was no significant change in VA of the biceps brachii in the immobilized arm, a finding that was contrary to our hypothesis. To our knowledge, only four studies have assessed changes in VA with immobilization in the upper limb, three of which revealed a decrease in VA with wrist immobilization (Clark et al., 2009, 2010, 2014), whereas one study of the elbow flexors did not observe such a change (Magnus et al., 2010). Therefore, we may have not observed a significant effect of immobilization on VA of the biceps brachii as corticospinal tract output from M1 is proportionally less toward proximal relative to distal muscles (Brouwer & Ashby, 1992). However, it is important to note that the study of the elbow flexors prior to our own merely suspended the arm using a sling rather than immobilizing the elbow joint, and furthermore, did not detect a significant change in elbow flexor strength following the intervention (Magnus et al., 2010). Changes in the ability to voluntarily activate skeletal muscles in response to disuse likely depends on factors beyond corticospinal tract input. For instance, activation of the muscles of the lower limb tends to depend less on input from the corticospinal tract compared to those of the upper limb (Brouwer & Ashby, 1990), and yet a recent investigation by MacLennan et al. (2021) revealed that VA of the quadriceps femoris decreased significantly after only 48 h of knee joint immobilization in young females. It has been suggested that maximal isometric torque, resting twitch torque, and VA can vary depending on whether the muscle is positioned in a more lengthened or shortened position during assessment in some, but not all individuals (Behrens et al., 2019; Prasartwuth et al., 2006). With our protocol, the elbow joint was fixed at an angle optimal for isometric flexion torque, but not resting twitch torque; as such, it may not have been sensitive enough to detect changes in VA within certain individuals (Prasartwuth et al., 2006).

The influence of muscle disuse on corticospinal excitability remains unclear; immobilization and bed rest studies have observed a decrease (Burianova et al., 2016; Karita et al., 2017; Okamoto et al., 2022), increase (Clark et al., 2008; Roberts et al., 2010; Zanette et al., 2004), or no change (Lundbye‐Jensen & Nielsen, 2008) in excitability. However, immobilization studies in which outcomes were measured following a shorter bout of immobilization (several hours to 4 days) have typically observed a reduction in excitability (Burianova et al., 2016; Karita et al., 2017; Okamoto et al., 2022). Therefore, in the present study we opted to measure changes in excitability at 24 h and 14 days post‐immobilization to evaluate the short‐ and long‐term effects of the intervention on corticospinal plasticity. We hypothesized a reduction in excitability at 24 h post‐immobilization; however, we did not observe such a change. This may be explained by the fact that proximal muscles such as the biceps brachii receive less corticospinal tract input relative to distal muscles such as those in the forearm and hand, which have been the primary sites of MEP recording in other upper limb immobilization studies (Clark et al., 2008; Kaneko et al., 2003; Karita et al., 2017). Otherwise, methodological differences in our study, such as extrapolating excitability as AUC rather than MEP amplitude obtained at a single intensity, may partly explain differences in findings between our study and those conducted prior. Altogether, while VA and corticospinal excitability are commonly applied to non‐invasively quantify neuromuscular function in vivo, findings from our study suggest that additional muscle and neural factors implicated in disuse‐induced functional decline should also be considered in future immobilization research studies; such factors may include adaptations in muscle tendon properties, sarcoplasmic reticulum function and motor unit behaviour (de Boer et al., 2007; MacLennan et al., 2021; Monti et al., 2021).

In the present study, we demonstrate changes in brain rs‐FC following upper limb immobilization in females. There is limited research on the influence of immobilization‐induced muscle disuse on FC. In alignment with previous research on brain activity and FC changes in response to limb immobilization, we suggest a potential involvement of the SMA, vPMC and cerebellum in muscle disuse‐induced neuroplasticity (Gandola et al., 2019; Garbarini et al., 2019). However, in contrast to our hypothesis, we did not observe any changes with respect to M1 nor S1 connectivity following immobilization. We initially hypothesized a functional segregation between the immobilized M1 and secondary motor areas based on a prior study by Newbold et al. (2020). Differences in study design may explain heterogeneity in the observed outcomes. We employed an elbow immobilization model using the non‐dominant arm of 12 female participants, and measured rs‐FC once pre‐ and once post‐immobilization. On the other hand, Newbold et al. immobilized the dominant arm and hand using a fiberglass cast; and, in favour of a more thorough assessment of the time course of rs‐FC changes, only included three participants, with fMRI scans performed daily before, during and after the 2‐week immobilization period. Due to the limited number of rs‐FC studies on upper limb immobilization, further research is needed to understand how upper limb disuse may induce functional reorganization of the brain.

Our findings highlight the potential involvement of the cerebellum in the neurophysiological response to muscle disuse, as its connectivity with various functionally related brain regions changed following immobilization. The cerebellum plays an essential role in movement amplitude, fluidity and the coordination of multi‐joint movements (Manto et al., 2012). Overall, changes in connectivity observed between the cerebellum and the left MTG, SMA and vPMC may suggest an influence of upper limb immobilization on movement planning, feedback and motor learning. The SMA for instance is responsible primarily for movement planning and communicates directly with the M1 (Nachev et al., 2008). The increase in connectivity between the bilateral SMA and the immobilized cerebellar lobule VIIIa may represent a compensatory mechanism in which the brain continues to plan movements that are typically performed with the immobilized arm. For example, the SMA is known to activate in response to interventions that attenuate strength decline during immobilization, such as during motor imagery training (imagining movement performance without explicit movement execution) (Jankelowitz & Colebatch, 2002). Unilateral muscle contraction has also been demonstrated to increase bilateral SMA and cerebellum activity, which may explain how unilateral strength training attenuates strength loss in an immobilized arm (Farthing et al., 2007; Sehm et al., 2010). In fact, unilateral strength training is known to exert a larger magnitude of effect when carried out during situations in which the untrained limb's function is physiologically restricted, such as in chronic stroke patients (Sun & Zehr, 2019), who have been demonstrated to experience overactivation of the SMA (Amengual et al., 2014). Therefore, an increase in functional connectivity between the SMA and cerebellum after 14 days of upper arm immobilization may represent an increase in the brain's sensitivity to preserve previously learned motor commands. To confirm this, further research with larger sample sizes is needed to determine whether a relationship exists between SMA–cerebellum connectivity and the capacity of the motor system to retain its ability to produce force from disused musculature, independent of changes in muscle size.

The MTG on the other hand is not a motor area; it is implicated in processing multimodal sensory feedback, particularly in the context of language, but has further been demonstrated to increase its connectivity with the cerebellum when detecting sensory mismatches following voluntary upper extremity movement (Van Kemenade et al., 2019). The decrease in connectivity between the immobilized cerebellar lobule VIIIa and the left MTG could signify a reduction in movement error detection capacity. Should this be true, it may partially explain how immobilization reduces joint coordination and complex motor task performance in the upper arm (De Marco et al., 2021; Moisello et al., 2008).

The vPMC works in conjunction with the motor system to play a role in error detection, particularly after movement execution (Garbarini et al., 2019). In opposition to the left MTG, the non‐immobilized vPMC underwent an increase in connectivity with the non‐immobilized cerebellar lobule VIIIb. Counteracting shifts in connectivity originating from the right and left cerebellum may suggest a reallocation of action‐feedback resources such that less attention is directed toward the disused arm. With wrist and hand immobilization, the immobilized vPMC has been demonstrated to undergo an increase in activity when attempting to close the fist of the immobilized hand immediately after cast immobilization, thus indicating successful error discrimination when comparing the intended movement to the outcome (Garbarini et al., 2019). But when the same task was attempted following a week of immobilization, the corresponding vPMC did not respond with any change in activity, reinforcing the possibility that action‐feedback mechanisms governed by the brain are reorganized once the brain understands that the immobilized limb cannot be used to produce certain movements (Garbarini et al., 2019).

We additionally observed a significant change in connectivity outside the cerebellum, in which the immobilized VLT and right posterior insula underwent a shift from negative to positive connectivity. The VLT, which is part of the motor thalamus, is thought to participate within a subcortical network involving the cerebellum and basal ganglia to control proprioception during movement (Bosch‐Bouju et al., 2013). Anatomical and experimental evidence has demonstrated that the VLT specifically facilitates the action of the cerebellum and basal ganglia on M1 (Middleton & Strick, 2000). The posterior insula is implicated in a variety of different functions, including somatosensory processing of information such as touch, pain and temperature, as well as interoception, which is the brain's awareness of the body's internal state (Uddin et al., 2017). In primates, the insula exhibits physiological connections with the thalamus, a logical finding as the processing of touch information is essential for proprioception (Jones & Burton, 1976). Because the thalamus and insula are seldom studied in conjunction, it is not clear what the significance is regarding the shift in connectivity between these two structures after immobilization. A prior fMRI study found that the posterior insula was not functionally connected to the thalamus at rest, whereas we observed a significant negative connectivity between these regions at baseline in our sample (Cauda et al., 2011). However, the authors of the previous study acknowledge that this finding is not necessarily consistent with other studies in primates revealing direct anatomical projections between these two brain regions (Cauda et al., 2011). Given the role of the posterior insula in perceiving changes within the body, its shift in connectivity with the VLT may be related to the detection of restricted mobility of the immobilized arm. The posterior insula has been demonstrated to activate in response to somatosensory input triggered by voluntary movement (i.e., moving a finger to touch an object), and has been further suggested to aid in the discrimination between somatosensory stimuli experienced by voluntary and externally induced limb movements (Limanowski et al., 2020). On the other hand, beyond serving as a relay point for movement execution carried out by M1, the thalamus has also been implicated in motor imagery, which involves movement intention as opposed to execution (Michelon et al., 2006). As mentioned prior, motor imagery training has been demonstrated to attenuate strength loss during limb immobilization (Clark et al., 2014). Therefore, in conjunction with the rs‐FC changes observed between the SMA and cerebellum, the shift toward positive connectivity between the posterior insula and the VLT may represent a compensatory mechanism in which the brain attempts to retain its capacity for movement planning and intention in response to a lack of sensory input derived specifically from movement of the immobilized arm. However, due to the limited number of studies regarding the clinical significance of these brain regions in the context of immobilization/muscle disuse, the interaction between the posterior insula and the VLT may be a mere response to the immobilization, rather than a compensatory mechanism. To test the above hypothesis, additional research is needed to determine whether the posterior insula and VLT are mechanistically involved in the efficacy of neural‐focused interventions for muscle strength loss during muscle disuse such as motor imagery training.

We did not observe any effect of immobilization on regional cortical thickness. With an immobilization period that was, on average, of similar duration, Langer et al. (2012) observed a decrease in motor cortical thickness in the hemisphere contralateral to the immobilized arm, accompanied by an increase in thickness in the homotopic region of the opposite hemisphere. The discrepancy between our findings and those of Langer et al. is likely due to differences in experimental design. Langer et al. specifically studied a sample of patients recovering from musculoskeletal injury of their dominant (right) arm. It has been shown previously that the functional detriments of immobilization are more pronounced in the dominant versus non‐dominant hand (Meugnot & Toussaint, 2015). Acute neuroplastic changes have additionally been found to be dependent not only on the disuse of the immobilized arm, but also on the use of the non‐immobilized arm, in the sense that significant changes occur only when the non‐immobilized arm is used sufficiently (Avanzino et al., 2011). In the event of dominant arm immobilization, the non‐dominant arm would likely experience an increase in use that is more notable than the dominant arm would with immobilization of the opposite arm, thus delaying or changing the nature of the resulting neurophysiological adaptations. In essence, the collective findings from our study and those of Langer et al. suggest that both functional and structural changes can occur in the brain during arm immobilization, and that the profile of these changes may depend on the circumstances related to the immobilization.

In conclusion, using criterion methods of measurement, we show that 14 days of upper limb immobilization induces a substantial decline in muscle strength of the elbow flexors and extensors in young females. Immobilization‐induced elbow flexor strength loss occurred even in the absence of significant elbow flexor muscle atrophy and was accompanied by functional changes in several brain regions implicated in movement planning and feedback and sensorimotor processing. Future research with larger, more diverse samples should aim to investigate the relative contribution of muscle atrophy, neurophysiological adaptations and other factors (e.g., tendon properties, muscle contractility) to disuse‐induced strength loss to facilitate the development of more holistic therapeutic approaches to preserve muscle function during obligatory periods of muscle disuse.

## AUTHOR CONTRIBUTIONS

Tyler A. Churchward‐Venne and Freddie Seo conceived and designed the research. Freddie Seo, Julien Clouette, Yijia Huang, Henri Lajeunesse, Frédérike Parent‐L'Ecuyer^1^, and Claire Traversa conducted the research. Freddie Seo, Julien Clouette, Yijia Huang, Caroline Paquette and Alexandra Potvin‐Desrochers analysed the data. Freddie Seo, Julien Clouette, Alexandra Potvin‐Desrochers Caroline Paquette and Tyler A. Churchward‐Venne interpreted the results of the experiments. Freddie Seo, Julien Clouette, Alexandra Potvin‐Desrochers, and Yijia Huang, prepared the figures and drafted the manuscript. Freddie Seo, Alexandra Potvin‐Desrochers, Caroline Paquette and Tyler A. Churchward‐Venne edited and revised the manuscript. All authors have read and approved the final version of this manuscript and agree to be accountable for all aspects of the work in ensuring that questions related to the accuracy or integrity of any part of the work are appropriately investigated and resolved. All persons designated as authors qualify for authorship, and all those who qualify for authorship are listed.

## CONFLICT OF INTEREST

None declared.

## Data Availability

Data described in the manuscript will be made available upon request pending application to and approval from the corresponding author.
